# Supplementary material for: The Small RNA Universe of Capitella teleta
Source: Front Mol Biosci. 2022 Feb 25;9:802814. doi: 10.3389/fmolb.2022.802814 (PMC8915122; doi:10.3389/fmolb.2022.802814)
Supplement: Supplementary file 1 [file DataSheet1.ZIP › Supplement/known/CAPTEscaffold_36111_47539.pdf]

Provisional ID	:	CAPTEscaffold_36111_47539
Score total	:	2334.6
Score for star read(s)	:	3.9
Score for read counts	:	2324.4
Score for mfe	:	1.7
Score for randfold	:	1.6
Score for cons. seed	:	3
Total read count	:	4571
Mature read count	:	4487
Loop read count	:	0
Star read count	:	84

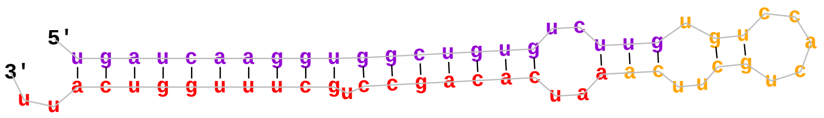

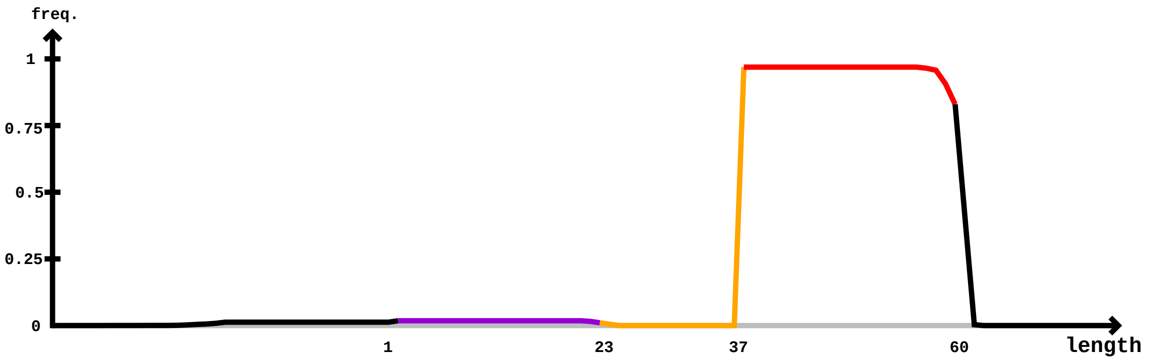

Star **Mature**

```
5'- cugcucuuccucacccucauugagaaccugcacuugaucaagguggcugugucuuguguccacugcuucaaaucacagccugcuuuggucauuugcugguuuuucauca   -3'   obs
    cugcucuuccucacccucauugagaaccugcacuugaucaagguggcugugucuuguguccacugcuucaaaucacagccugcuuuggucauuugcugguuuuucauca         exp
    cugcucuuccucacccucauugagaaccugcacuugaucaagguggcugugucuuguguccacugcuucaaaucacagccugcuuuggucauuugcugguuuuucauca         known
    ....................(((((((.(((..(((((((((((((((((..(((.((.....))..)))..))))))).))))))))))..))).))))))).....   reads   mm   sample
    ....ucuuccucacccucauugagaaccugcacu...........................................................................   1       0    seq
    ........ccucacccucauugagaaccugcacu...........................................................................   1       0    seq
    ...........cacccucauugagaaccugcacu...........................................................................   2       0    seq
    ............acccucauugagaaccugcacu...........................................................................   7       0    seq
    .............cccucauugagaaccugcacu...........................................................................   9       0    seq
    ..............ccucauugagaaccugcacu...........................................................................   7       0    seq
    ...............cucauugagaaccugcacu...........................................................................   12      0    seq
    ................ucauugagaaccugcacu...........................................................................   20      0    seq
    ..................................ugaucaagguggcugugucu.......................................................   11      0    seq
    ..................................ugaucaagguggcugugucuu......................................................   25      0    seq
    ..................................ugaucaagguggcugugucuAg.....................................................   1       1    seq
    ..................................ugaucaagguggcugugucuug.....................................................   43      0    seq
    ..................................Agaucaagguggcugugucuug.....................................................   1       1    seq
    ..................................ugaucaaggAggcugugucuug.....................................................   1       1    seq
    ..................................ugaucaagguggcugugucuugA....................................................   2       1    seq
    ......................................................................aaucacagccugcuuuggu....................   19      0    seq
    ......................................................................aaucacagccugcuuugguc...................   35      0    seq
    ......................................................................aaucacagccugcuuAgguca..................   1       1    seq
    ......................................................................aaucacagccugcuuuggucа..................   233     0    seq
    ......................................................................aaucacagccugcuuugguAa..................   2       1    seq
    ......................................................................aauUacagccugcuuugguca..................   1       1    seq
    ......................................................................Uaucacagccugcuuuggucau.................   1       1    seq
    ......................................................................aaucacagccugcuuuggucau.................   349     0    seq
    ......................................................................aaucacagccugcuuugguGau.................   1       1    seq
    ......................................................................Gaucacagccugcuuuggucau.................   1       1    seq
    ......................................................................aaucacagccugcuuuAgucau.................   2       1    seq
    ......................................................................aaucacagccugcuuugguAauu................   3       1    seq
    ......................................................................Naucacagccugcuuuggucauu................   3       1    seq
    ......................................................................aaucacagccugcuuugAucauu................   1       1    seq
    ......................................................................Gaucacagccugcuuuggucauu................   1       1    seq
    ......................................................................aaucacagccugcuuuggucauG................   5       1    seq
    ......................................................................aaucacagccugcuuugUucauu................   2       1    seq
```

```
                                  Star                                Mature
cugcucuuccucacccucauugagaaccugcacuugaucaagguggcugugucuuguguccacugcuucaaaucacagccugcuuuggucauuugcugguuuuucauca
.....................................................................aaucacagccugcuuuAgucauu................    11      1      seq
.....................................................................aaucacagccugcuuuggucGuu................    1       1      seq
.....................................................................aaucacagccugcuuuggucaAu................    3       1      seq
.....................................................................aaucacagccugcuuCggucauu................    2       1      seq
.....................................................................aauAacagccugcuuuggucauu................    3       1      seq
.....................................................................aaucaUagccugcuuuggucauu................    1       1      seq
.....................................................................aaucacagccAgcuuuggucauu................    2       1      seq
.....................................................................aaucacagccugcuuuggucauA................    13      1      seq
.....................................................................aaucacagcAugcuuuggucauu................    1       1      seq
.....................................................................aaucGcagccugcuuuggucauu................    1       1      seq
.....................................................................aaucacagccugcuuGggucauu................    1       1      seq
.....................................................................aaucacagccugcuAuggucauu................    1       1      seq
.....................................................................aaucacagccugcuuugguUauu................    1       1      seq
.....................................................................aaucacagccugcuuuggucauC................    3       1      seq
.....................................................................aaAcacagccugcuuuggucauu................    7       1      seq
.....................................................................aaucacagccuUcuuuggucauu................    2       1      seq
.....................................................................aaucUcagccugcuuuggucauu................    1       1      seq
.....................................................................aaucacagAcugcuuuggucauu................    1       1      seq
.....................................................................aaucacagccugcuuuggucauu................    3706    0      seq
.....................................................................aaucacagccugGuuuggucauu................    1       1      seq
.....................................................................aaucacagccugcuuAggucauu................    2       1      seq
.....................................................................aaucaGagccugcuuuggucauu................    1       1      seq
.....................................................................aaucacagccugAuuuggucauu................    3       1      seq
.....................................................................aaucacagccugcCuuggucauu................    2       1      seq
.....................................................................aaucacagccugcuuuggucauuu...............    32      0      seq
.....................................................................aaucacagccugcuuuggucauuC...............    5       1      seq
.....................................................................aaucacagccugcuuuAgucauuu...............    1       1      seq
.....................................................................aaucacagccugcuuuggucauuA...............    4       1      seq
.....................................................................aaucacagccugcuuuggucauuAg..............    1       1      seq
.....................................................................aaucacagccugcuuuggucauuuU..............    14      1      seq
```
